# Above and Below the Diaphragm: A Previously Undescribed Case of Recurrent Boerhaave Syndrome Diagnosed With Computerized Tomography Esophagram

**DOI:** 10.7759/cureus.24015

**Published:** 2022-04-10

**Authors:** Rohan Anand, Yana Puckett, Catherine A Ronaghan

**Affiliations:** 1 Surgery, Texas Tech University Health Sciences Center, Lubbock, USA; 2 Surgery, West Virginia University School of Medicine, Charleston, USA

**Keywords:** stenting, ct esophagram, etiology and pathogenesis, esophageal rupture, boerhaave's syndrome

## Abstract

Boerhaave syndrome, defined as a spontaneous rupture of the esophagus, is an uncommon clinical entity. Recurrent spontaneous rupture of the esophagus is even rarer and has only been described in a handful of case reports. The rupture most often occurs in the thoracic esophagus. Spontaneous rupture of the intraabdominal esophagus is extremely rare.

The extravasation of gastric contents, including bile, acid, and bacteria, into a body cavity precipitates severe sepsis. This results in a high mortality rate without emergent treatment. Such treatment often necessitates surgical repair with primary closure, tissue grafts, or esophagectomy in particularly severe cases.

This is a case of a 64-year-old male who suffered Boerhaave syndrome twice separated by two years. The patient was transferred from an outside facility initially presenting with chest and abdominal pain, which developed after eating. CT esophagram with water soluble contrast demonstrated contrast extravasation into the right mediastinum/hemithorax, consistent with a diagnosis of Boerhaave syndrome. Repair was accomplished with an intercostal muscle pedicle patch, and the patient was subsequently discharged. This case report details, to our knowledge, the first case of a left intraabdominal and right thoracic esophageal rupture combination.

## Introduction

Esophageal rupture is a rare clinical condition with an estimated annual incidence of 3.1 per million [1]. The most common cause is iatrogenic perforation due to instrumentation. Boerhaave syndrome, defined as spontaneous rupture of the esophagus, while the next most common cause of esophageal rupture, accounts for only 15% of all cases [2]. The condition was first described by Dutch physician Herman Boerhaave in the 18th century after whom the condition is named. This is most often due to a sudden increase in intraluminal esophageal pressure in addition to the normal negative pressure of the thoracic cavity, causing a transmural tear in the esophageal wall. Causes include Valsalva maneuver, swallowing food, and prolonged cough. Vomiting against a closed glottis, which is more likely to occur in an inebriated patient, is particularly classic. The resulting extravasation of bile, gastric acid, and food particles into the mediastinum, pleural space, and abdominal cavity is largely responsible for the rapid sepsis that confers a high mortality rate. While a rupture may occur anywhere along the length of the esophagus, it most commonly occurs in the left posterolateral distal esophagus.

Recurrent Boerhaave syndrome is extremely rare and has only been described in a handful of cases in the literature [3-9]. Intraabdominal rupture of the esophagus secondary to Boerhaave syndrome is even rarer, and has only been described in patients as an isolated occurrence in their lifetime [10]. This case describes both an intraabdominal esophageal rupture and a thoracic esophageal rupture two years later, which to our knowledge has not been previously reported in the literature.

## Case presentation

**Figure 1:**
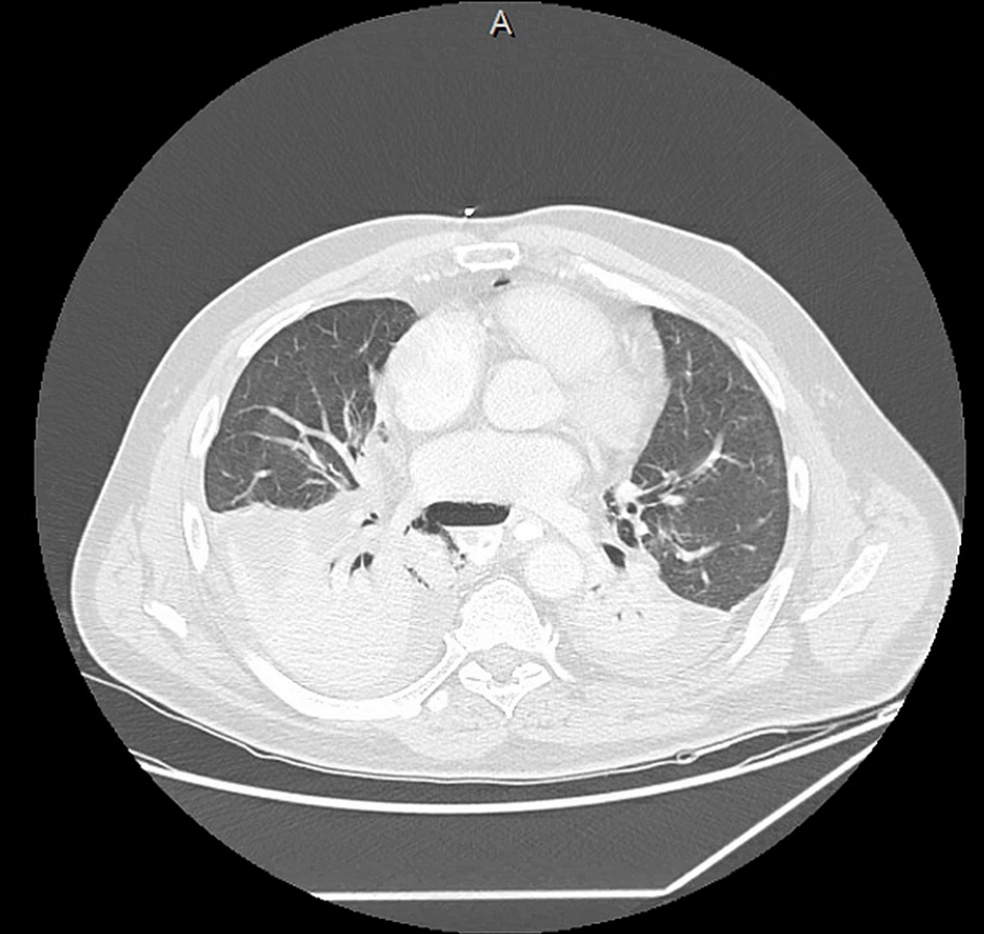
CT chest demonstrating extravasation of oral contrast from esophagus into the right mediastinum/hemithorax. Also note pneumomediastinum, bilateral pleural effusions, and consolidations

**Figure 2:**
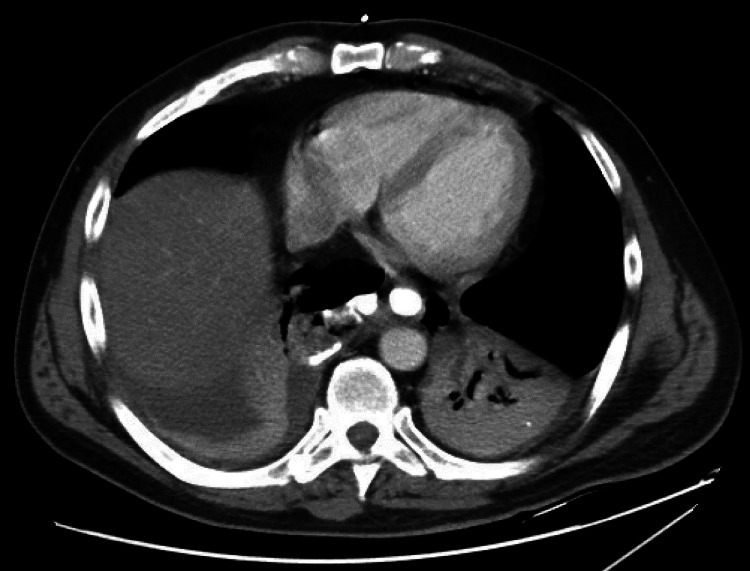
CT chest demonstrating extravasation of oral contrast from esophagus into the right mediastinum/hemithorax with associated pneumomediastinum

**Figure 3:**
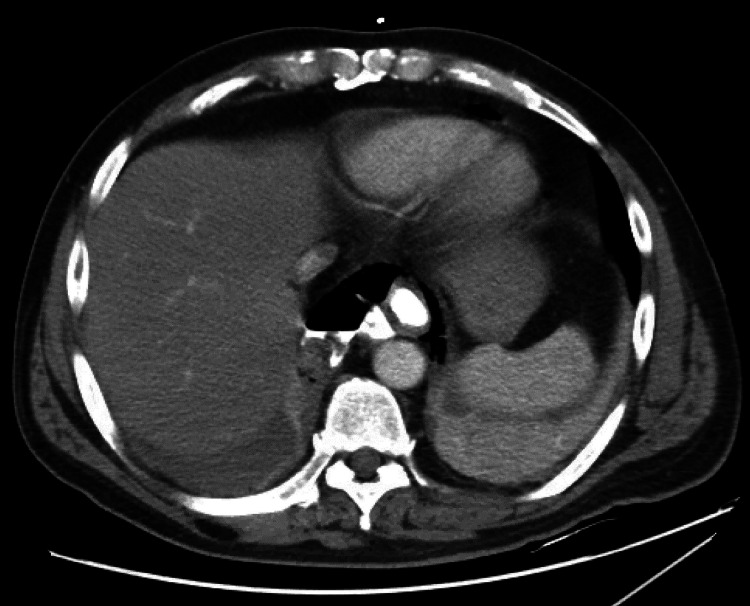
Continuation of CT in Figure 1, with better demonstration of extravasated oral contrast and air within the mediastinum

A 64-year old male with a past medical history of alcohol abuse was transferred to our facility after the acute onset of chest and abdominal pain after eating food. He was intubated in transit and hemodynamically stable upon arrival. Workup at the outside hospital demonstrated diffuse mediastinal air and bilateral pleural effusions. Labs were notable for a WBC of 16,470 cm^3^. Arterial blood gas (ABG) was notable for pH of 7.163 and partial pressure of carbon dioxide (pCO2) of 63.4, indicating acute respiratory failure. A nasoesophageal tube was placed to facilitate imaging which included CT esophagram using water soluble contrast (WSC). This demonstrated bilateral pleural effusions and contrast extravasation into the right mediastinum/hemithorax, consistent with a distal esophageal rupture (Figures 1-3). Of note, the patient’s surgical history included repair of an intraabdominal left sided spontaneous esophageal rupture two years earlier via a midline laparotomy. This interestingly resulted in an incisional hernia with loss of domain noted at presentation to our facility.

Cardiothoracic surgery was consulted, and the patient was emergently taken to the operating room. A right posterolateral thoracotomy was performed, and the chest was entered at the seventh intercostal space. Clear brown fluid was encountered, with 700-800 ml initially aspirated from the pleural space. A 2-cm transmural tear was identified at the right esophagus immediately above the diaphragm. Due to the soft and edematous esophageal tissue, suture repair was deferred in favor of an intercostal muscle pedicle patch. A 28 French chest tube was used to drain the area adjacent to the esophageal tear. Additionally, a 32 French chest tube was placed in the right costophrenic gutter, and a second 32 French chest tube drained the chest apex.

**Figure 4:**
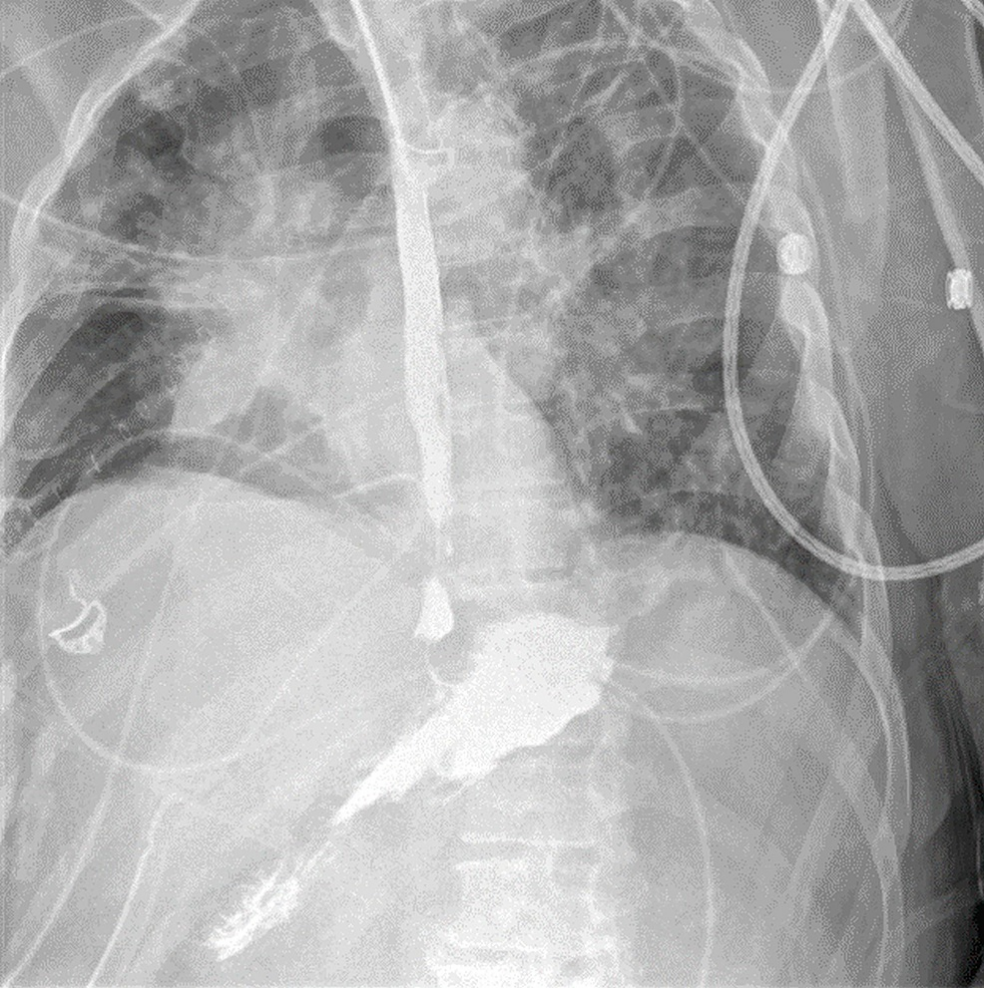
Contrast esophagram on POD 19 demonstrating retention of contrast in the esophagus and stomach with no extravasation into the pleural cavities or mediastinum

The patient had a prolonged postoperative course in the SICU. This included episodes of atrial flutter and fibrillation requiring amiodarone. Fluid cultured from the right pleural space grew *Capnocytophaga sputigena* on post-operative day (POD) 17, for which a therapeutic course of antibiotics based on sensitivities was completed. Repeat esophagogram with WSC on POD 19 demonstrated no extravasation (Figure 4) and repeat EGD on POD 25 revealed complete healing of the esophagus. The remainder of the hospital course was uncomplicated, and the patient was discharged on POD 34.

## Discussion

Early recognition of Boerhaave syndrome is critical to decreasing morbidity and improving survival. The classic Mackler’s triad of vomiting, severe chest pain, and subcutaneous emphysema, while highly specific of Boerhaave syndrome, is present in only 14-30% of patients [11]. A thoracocentesis or chest tube placed for symptomatic relief that drains green or brown acidic fluid, contains food particles, or has an elevated amylase level is also suggestive. Additional symptoms are often nonspecific, including dysphagia, odynophagia, dyspnea, fever, and tachycardia.

Imaging is key to confirmation of esophageal rupture. The diagnostic standard is CT of the chest, abdomen, and pelvis [12]. CT esophagram utilizing WSC aids in localization of the rupture, specifically, location in relation to the diaphragm and lateralization. In this case, a nasoesophageal tube was positioned in the proximal esophagus for instillation of WSC immediately prior to imaging. This effectively created a CT esophagram which lateralized the site of the rupture in the setting of bilateral pleural effusions. This proved particularly useful as the CT esophagram revealed a right distal thoracic esophageal rupture, an uncommon occurrence in Boerhaave syndrome.

Prior to the accessibility of CT scans, other imaging modalities such as CXR and contrast fluoroscopic esophagram were readily used for diagnosis. CXR may reveal several suggestive diagnostic clues, including widened mediastinum, pleural effusion ipsilateral to the site of rupture, and air dissection into the pleural, mediastinal, or subdiaphragmatic space. It is important to note that CXR may be normal in early cases [2,11]. Contrast fluoroscopic esophagram used to be the gold standard for diagnosis of esophageal rupture, but has since been replaced with CT.

While esophageal rupture presenting with small tears and a mild clinical course may be candidates for conservative treatment, most cases require surgical intervention. Options for repair of the rupture include single- or double-layer primary closure. Reinforcement options include tissue grafts (pleural flaps, diaphragmatic pedicle grafts) and muscle flaps to reduce the risk of postoperative leak [2,12]. Esophagectomy may be necessary for particularly severe rupture or the presence of additional underlying esophageal pathology. The advent of endoscopic esophageal stenting has shown promising results in specific scenarios. A pooled analysis of 25 studies by Boeckel et al. showed healing of the rupture in 85% of patients that underwent stent placement for esophageal rupture [13].

Recurrent Boerhaave syndrome is exceedingly rare. Our literature review identified eight published cases, all in the thoracic esophagus. This case study is unique for several reasons: a left-sided intraabdominal rupture followed two years later by a right-sided thoracic rupture. Ruptures involving the left distal thoracic esophagus occur in 65-90% of cases, with right or intraabdominal esophageal ruptures being extremely rare [14,15]. A number of theories have been proposed to explain this occurrence. The esophageal wall is relatively unsupported by abdominal viscera in the lower left relative to the right. Another theory suggests the difference in structure of diagonal and circumferential fibers of the esophagus playing a role. A more contested theory involves penetration of vessels and nerves in the wall of the lower esophagus [11,14].

Interestingly, in the reported recurrent thoracic Boerhaave syndrome cases, five of the eight cases involved right-sided esophageal ruptures, but none involved an intraabdominal rupture. Intraabdominal esophageal ruptures have only been described in the literature six times; none of these cases involved an additional esophageal rupture [10]. A case of recurrent Boerhaave syndrome in which one of the ruptures occurred in the intraabdominal esophagus and one in the thoracic esophagus has not been previously reported in the literature.

## Conclusions

Localizing the site of esophageal rupture in patients with suspected Boerhaave syndrome can be challenging when bilateral pleural effusions are present. This case demonstrates the usefulness of CT esophagram utilizing WSC, as the patient had bilateral pleural effusions at presentation. In this scenario, the CT esophagram provided very important information as to the lateralization of the much rarer right sided thoracic esophageal rupture.

Recurrent Boerhaave syndrome is extremely rare, with only a few cases reported in the literature. This is the first case report of a left intraabdominal and a right thoracic rupture of the esophagus combination, which occurred within a two-year interval.
